# The Structure and Functions of the Brain

**Published:** 1859-12

**Authors:** C. A. Hartmann


*The Structure and Functions of the Brain.*

(From the *Cleveland Medical Gazette.*)

Dr. Luigi Meschi (Gazz. Sarda, 1—22, 1857,) offers the following views touching that most interesting, and still little explored, field of medical investigation, the brain and its physiology.

According to the difference in the cerebral structure, the entire animal kingdom is to be classified as follows: 1. Ganglia forming the termination of centripetal and centrifugal nervous fibres, as in the Acalephs and Helminthes. 2. One or more central ganglia, receiving, besides their own ramifications, fibres from other ganglia, forming in that way a two-fold system: peripheric and central, or sympathetic and cerebral ganglia. The number of the former increases with the development of the latter; the extreme is reached in a single part of central ganglia. Molluscs, Cetacea. 3. Two pairs central ganglia: an anterior and a posterior one, olfactory and optic, with hemispheres between them, and another ganglia, the cerebellum, resting on the spinal marrow. Fishes, Reptiles, Birds. 4. Cerebral ganglia covered by the hemispheres, for both of which the names of enveloped and enveloping ganglia are próposed. The Corpus Callosum only unites the hemispheres, and has not the specific properties of a ganglion. The cerebellum exhibits a similar arrangement: the two hemispheres, connected with each other by the pons Varoli, vermis superior and vermis inferior, cover the corpora rhomboidea as interior ganglia. The corpora quadrigemina, possessing the character of both the forms of ganglia already described, and exchanging fibres with the thalamus as well as with the cerebellum, are to be considered as intermediate ganglia.

There is a current in the nervous fibres, but that in the sensitive nerves moves in an opposite direction to the one in the motory nerves. The current of the sensitive nerves either reaches the brain and is there perceived, or it is diverted by a ganglion in its way and transported to motory fibres, for the ganglia manifests an attractive as well as a repulsive action on the nervous current, combining a centripetal and a centrifugal pole analagous to the positive and negative electrical poles, the one connected with the sensitive, the other with the motory conductor. But both these poles are also found in the nervous fibres, be these sensitive or motory, and what is true in electricity, also applies here: The opposite poles attract each other, etc.

Now, with the centripetal current three things may happen: 1. If there is no ganglia in the way, the current reaches the brain and is felt; or, 2, the current meeting one or several ganglia is forced by them upon the motory fibres, and therefore not felt; or, 3, a part only of the current is deviated by the ganglia, while the other part goes on to the brain and produces there a sensible impression, although in a slighter degree. This explains, why the vegetative organs, dependent upon the sympathetic nerve, are insensible as long as nothing unusual takes place; these very organs, however, send a very perceptible current to the brain, as soon as any irritation increases the strength of the current; inflammation of the stomach or bladder, for instance, produces such an increase, apparently by increasing the energy of the nerves.

The principle working in the nerves results in some way from the blood; at least we have to suppose this, in order to explain the effects of the nerves that are connected with the heart. Cutting the vagi, we suppress all operation exercised on the heart by the fibres of the accessory nerve and surrender that organ to the influence of the sympathetic fibres from the lower ganglia of the neck. The nervous fluid, no longer allowed to extend its motions through the spinal marrow and the brain, is concentrated in those sympathetic fibres, and consequently the energy of the heart is increased, at least for some time. Under the influence of the accessorius alone, the cardiac ramification of the sympathetic nerve being separated, the motions of the heart are, on the contrary, diminished in frequency as well as force, for obvious reasons.

The brain, forming merely a combination of ganglia, cannot possess sensibility or motory influence any more than the ganglia. The brain as a whole, is in that respect even less powerful than the ganglia, and what little of sensitive influence is allowed to pass to the brain, is soon spent in the central parts of it, so that little or nothing of it is left to spread over the cerebral convolutions. The effect of the sensitive currents on the brain thus being destroyed, certainly no motory impetus can result from them. To explain, therefore, the many motory effects evidently emanating from the brain, the adoption of another principle, working on the brain, is inevitable. This principle is the soul, the great cause of all arbitrary animal action, free, conscious, intelligent. The soul resides principally in the convolutions of the brain, and her action is manifested in four forms: consciousness, instinct, intellectuality and arbitrariness. The soul is the independent operator, and the ganglia, with their more automatic influence, produce in their combination every manifestation of animal life.

We cannot follow the author any further in the particulars of his explanation, nor do we at present feel inclined to enter into any special criticism of his doctrines. Waiting for the result of more extensive explorations, now instituted, and based upon skilful experiments, we do not deem it worth while to refute at length the errors committed, either by a perverse application of facts, or by illogical and unscientific conclusions drawn from correct observations; especially in reference to the vital action of the brain. While we may agree with Dr. Meschi in taking the brain as a more developed, or rather more highly organized ganglion, we must draw, from this very supposition, the inference of a more extended power in the central parts of the nervous system. Why is a man's intellect disturbed even to raving madness, by the smallest piece of bone pressing the brain, or by a diseased condition of that organ, if the soul as independent source of the intellect, is not the expression of the vital energy of the brain? That wonderful complex of nervous structures is certainly not the mere residence of the soul, enclosing it just like the shell that is secreted from, and formed around, the body of the snail. All the facts that we have, bearing upon the relation existing between brain and intellect or soul, rather lead us to the conclusion of the brain being the basis and the source of every intellectual action. The explorations already referred to will, undoubtedly, give us some more light upon this highly important point, and we shall then, probably, find an occasion to express our own views more fully, and to canvass the whole matter more thoroughly.

C. A. Hartmann.

